# Man Down Situation Detection Using an in-Ear Inertial Platform

**DOI:** 10.3390/s21051730

**Published:** 2021-03-03

**Authors:** Alex Guilbeault-Sauvé, Bruno De Kelper, Jérémie Voix

**Affiliations:** Université du Québec, École de technologie supérieure (ÉTS), Montréal, QC H3C 1K3, Canada; alex.guilbeault-sauve.1@ens.etsmtl.ca (A.G.-S.); bruno.dekelper@etsmtl.ca (B.D.K.)

**Keywords:** man down, fall detection, worker safety, monitoring, inertial platform, wearable sensors

## Abstract

Man down situations (MDS) are a health or life threatening situations occurring largely in high-risk industrial workplaces. MDS automatic detection is crucial for workers safety especially in isolated working conditions where workers could be unable to call for help on their own, either due to loss of consciousness or an incapacitating injury. These solution must be reliable, robust, easy to use, but also have a low false-alarm rate, short response time and good ergonomics. This project aims to improve this technology by providing a global MDS definition according to a combination of three observable critical states based on characterization of body movement and orientation data from inertial measurements (accelerometer and gyroscope): the worker falls (F), worker immobility (I), the worker is down on the ground (D). The MDS detection strategy was established based on the detection of at least two distinct states, such as F-I, F-D or I-D, over a certain period of time. This strategy was tested using a large public database, revealing a significant reduction of the false alarms rate to 1.1%, reaching up to 99% accuracy. The proposed detection strategy was also incorporated into a digital earpiece, designed to address hearing protection issues, and validated according to an *in vivo* test procedure based on simulations of industrial workers normal activities and critical states.

## 1. Introduction

Certain areas of industrial workplaces, like mining, forestry, construction and fire-fighting, are known as precarious and dangerous, involving numerous physical and mechanical hazards as well as lone-work situations, where accidents and morbidity are more frequent. Labour laws require employers and industries to ensure employee protection by adopting preventive measures, appropriate safety equipment and occupational health and safety training. However, any given workplace will never be totally safe from accidents especially for a lone worker and high-risk workers. In this context, portable devices alerting a control center when an emergency is detected should be worn with main advantage to call help when worker is unable to do it on his own, either due to loss of consciousness or an incapacitating injury. These systems are therefore essential in ensuring occupational health and safety in the workplace, and their reliability is just as critical. Solutions must also meet industry requirements in terms of reliability, robustness and ease of use, as well as featuring low false-alarm rate, short response time and good ergonomics. Poor designs could result in additional costs for employers, loss of confidence in the technology and lesser deployment of this technology in the industry.

According to the IRSST, falls from heights, from same level or from slips constitute the greatest causes of occupational injuries, responsible for more than 21% during 2010–2012 [1]. Compensation paid for victims of injuries in case of falls from heights are larger than the average and constitute a significant risk of decreased productivity and quality of life [2]. Many existing devices are designed to detect only falls, according to the 327 studies conducted up to 2013 [3], and available solutions has aimed towards the elderly-care market since the elderly are vulnerable and most prone to fall. Several solutions use subject post-fall disability state, mostly characterized by immobility or down position state, to limit detection errors whenever the device fails to detect a fall occurrence, making it a very important aspect that should be included in a robust fall detection solution [3]. Moreover, the post-fall disability state duration is a direct factor of fall severity, weakness of the victims and mortality rate [3,4].

The NSERC-EERS Industrial Research Chair in In-Ear Technologies (CRITIAS), who has developed a unique technology designed to protect industrial workers from noise-induced hearing loss, kickoffs this project to integrate a man down situations (MDS) detection solution into a digital earpiece prototype by incorporating an inertial platform and addressing both issues with a single and simple solution. While some consumer MDS detection devices have recently been developed for elders using hearing-aid devices [5], there has been very few scientific studies on MDS detection usage in workplace. Moreover, MDS definition is not consistent through studies, distinguishing types of emergencies such as falls, dangerous substance exposure, health problems (stroke, incidents, heart attacks) or loss of consciousness [6], which some lead to more complex solutions, as vital signs monitoring (respiration, heart rate and galvanic skin response sensors) and several environmental hazards detection (gas, chemicals, noise).

Without state-of-the-art scientific definition of man down situations, this project seeks a global and simple detection solution based on characterization of motion and orientation tracking using an in-ear inertial platform, for all emergencies faced by workers, where nature and causes of danger are innumerable, diverse and hard to predict considering all variables like workplace, work tasks, workers health, physiognomy, etc. The detection strategy and digital earpiece solution implementation will be validated using test scenarios inspired by typical activities performed by targeted workers.

## 2. Materials and Methods

### 2.1. Motion and Orientation Tracking

The motion and orientation tracking methodology is based on an inertial measurement unit (IMU), which has a 3-axis accelerometer, for linear acceleration measurements *a* = ${\left[a_{x}\;a_{y}\;a_{z}\right]}^{T}$, and a 3-axis gyroscope, for rotational speed measurements ω = ${\left[\omega_{x}\;\omega_{y}\;\omega_{z}\right]}^{T}$. Inertial sensors are affected by numerous measurement errors such as constant error sources due to cross axial coupling, scaling factors, orthogonal axis misalignment and measurement biases [7], and continuous errors that evolve over time due to random noise processes, including numerical quantification, random gyroscope angle walking, continuous random walk, bias stability, and continuous measurement drift [8]. Constant error sources are handled with unique static calibration while continuous errors are compensated with dynamic calibration over time. The iterative least-squares method proposed by [9] was used for acceleration measurement calibration since it does not require any external equipment and based on a large acceleration data set of multiple sensor positions. Since the direction and magnitude of the Earth’s gravity is known and constant, the compensation coefficients of the accelerometer model can be determined to correct acceleration vector norm that should ideally represent a unitary sphere centered at the origin. The rotational speed instantaneous bias is corrected firstly by subtracting the average rotational speed offset while the gyroscope is stationary (ω=0). Then, correction of rotational speed bias drift is proceed by integrating the gyroscope’s rotational errors with respect to the product of both inertial sensor measurements and their fusion [10]. The optimized gradient method from [10] is used to find an optimal orientation estimation, given in quaternion representation, which is a mathematical entity *q* = ${\left[q_{1}\;q_{2}\;q_{3}\;q_{4}\right]}^{T}$ simplifying rotation calculation in space and avoiding the singularity problems of trigonometric functions [11,12].

### 2.2. *SisFall* Database

The large public database *SisFall* includes 4510 inertial data records of various scenarios of activities of daily living (ADL) and falls [13]. The test measurements were captured using an inertial platform, two accelerometers and a MEMS-type gyroscope, placed on the waist belt of participants. Only data from the Freescale MMA8451Q 3-axis accelerometer (14 bits, ±8 g) and the InvenSense ITG-3200 3-axis gyroscope (16 bits, $\pm 2000^{\circ }$/s), with a 200 Hz sampling rate, are used in this work.

Since the database was specifically intended to classify fall events, body movements making up the MDS are not all represented, however it is an excellent data source to characterize rigorously the critical states and MDS, at the heart of the detection strategy.

### 2.3. Features Characterization

The IMU provides raw orientation and displacement data from which are extracted the specific movement features that are relevant to MDS detection. The main difficulty is to discriminate movement features relevant to MDS from natural movement features coming from a wide variety of work environment activities, such as discriminating a critical fall from bending over to pick up an object on the ground, etc. In most cases, the extreme nature of the movements at play in a MDS is the most revealing feature as compared to the movements in play in normal work activities. Hence, the extrema extracted from the signals provided by the IMU have been chosen as the main feature for MDS detection.

Inertial data are processed in order to extract relevant physical signals for critical states detection, such as the acceleration norm A(t), the rotational speed norm W(t), the tilt angle ρ and its derivative $\dot{\rho }\left(t\right)$.

(1) $$A\left(t\right)=\left||a\left(t\right)|\right|=\sqrt{a_{x}^{2}\left(t\right)+a_{y}^{2}\left(t\right)+a_{z}^{2}\left(t\right)}$$

(2) $$W\left(t\right)=\left||\omega \left(t\right)|\right|=\sqrt{\omega_{x}^{2}\left(t\right)+\omega_{y}^{2}\left(t\right)+\omega_{z}^{2}\left(t\right)}$$

(3) $$\rho =\arccos\left(\frac{g\cdot v}{\left||g||||v|\right|}\right)=\arccos\left(g\cdot v\right),v=q^{*}gq$$

(4) $$\dot{\rho }\left(t\right)=d\rho \left(t\right)/dt$$

The tilt angle ρ is obtained by calculating the angle between the orientation vector *v*, from the orientation estimation as a quaternion *q*, and the vertical axis represented by the gravity vector *g*. The characterization of the feature signals is based on their extreme values analysis establishes statistical models serving as a basic index of detection probability for each critical state. The statistical models are built based on extreme values distribution of the temporal maximum and minimum of the mean or variance of feature signals, segmented according to different time windows, namely extreme value signals. Depending on the nature of the critical states, specific sets of extreme value signals are analyzed on occurrences of the critical states from the reference dataset.

The extreme values are then characterized according to two probability distribution models. First, the normal distribution $N(\mu ,\sigma^{2})$, describing random events of natural phenomena, with a probability density function of a random variable *X* given by

(5) $${\mathrm{pdf}}_{\mathrm{norm}}\left(X\right)=\frac{1}{\sigma \sqrt{2\pi }}\exp\left(-\frac{{\left(x-\mu \right)}^{2}}{2\sigma^{2}}\right),x\in R$$

where μ is namely the mean and σ the standard deviation.

Then, the Gumbel distribution G(u,β), also known as the generalized extreme value distribution of type I (k=1), commonly used to predict rare events or extreme values of normal-type or exponential initial distribution data [14]. The probability density function is given by

(6) $${\mathrm{pdf}}_{\mathrm{gumbel}}\left(X\right)=\frac{1}{\beta }\exp\left(-\frac{\left(x-u\right)}{\beta }\exp\left(-\frac{\left(x-u\right)}{\beta }\right)\right),x\in R$$

where *u* is namely the distribution locality and β the scale, estimated by resolving the equation system based on maximum likelihood method [15] with β>0.

In practice, Gumbel and normal distributions may have similar appearance, therefore, it is possible to discriminate between the two distributions by using the Ratio of Maximized Likelihood (RML) as test statistic. As such, the logarithm of the RML Function for the normal distribution over the Likelihood Function of the Gumbel distribution with a value greater than zero mandate the use of the normal distribution, and otherwise, the Gumbel distribution [16].

### 2.4. Detection Theory

The present study focuses on binary statistical test, also binary classification theory, which defines a mathematically formalized decision-making method based on known statistical models in order to make a predictive decision using an independent data set. The null hypothesis $H_{0}$ defines the decision that the event did not occur and the alternative hypothesis $H_{1}$ as the decision that the event did occur. The probability rates of event detection $P_{D}$ when the event actually occurred and the probability rate of a false alarm $P_{\mathrm{FA}}$, also known as the type I error, are defined by the following equations:

(7) $$P_{D}=\mathrm{Pr}\left\{H_{1}|H_{1}\right\}$$

(8) $$P_{\mathrm{FA}}=\mathrm{Pr}\left\{H_{1}|H_{0}\right\}$$

The detection performance is calculated according to the number of positive and negative results of detection as well as by their classification as “true positive” (TP), “false positive” (FP), “true negative” (TN) and “false negative” (FP) as follows their true classification. The accuracy indicates the detection behavior by evaluating the results of true predictions without considering the classification of the tests.

(9) $$Accuracy=\frac{\mathrm{TP}+\mathrm{TN}}{P+N}$$

The Matthews correlation coefficient (MCC) is commonly used to evaluate the performance of predictive models, especially in personalized medicine (genetic testing, molecular analyzes, etc.), and represents a discretization of Pearson correlation for binary classification of two distinct groups. The MCC given by

(10) $$\mathrm{MCC}=\frac{\left(\mathrm{TP})(\mathrm{TN})-(\mathrm{FP})(\mathrm{FN}\right)}{\sqrt{\left(\mathrm{TP}+\mathrm{FP})(\mathrm{TP}+\mathrm{FN})(\mathrm{TN}+\mathrm{FP})(\mathrm{TN}+\mathrm{FN}\right)}}$$

reflects better evaluation of detection performance over accuracy and is considered as a robust and reliable statistical measure with the ability to truthfully bring out any prediction deficiencies from dataset by providing a more complete and informative response [17]. In this study, the MCC is used to determine optimal time window sizes and critical states detection thresholds, instead of the area under the curve (AUC) of the receiver operating characteristic (ROC) curve which have some drawbacks and need the complete analysis of $P_{D}\in \left(0,1\right)$ range.

### 2.5. Man Down Situation Definition

For the purpose of MDS detection, a global definition is proposed according to the observation of three distinct critical states, namely the Immobility state (I), the Fall state (F) and Down position state (D). The combination of these critical states describes most of emergencies faced by workers in industrial workplaces. In this study, the fall state is defined as the falling phase pre-impact, characterized by a free fall and a large variation of the inclination of the body, and the fall-impact phase, which is characterized by a great force resulting from the collision of the body with either the ground or another object. The immobility state is defined as a low level of movement of the worker’s body during a significant time period. Finally, the down position state is simply defined by the near horizontal body’s angle.

The proposed hypothesis is that fusion of these three critical states enables a more accurate and reliable MDS detection. More specifically by looking for multiple combinations of concurrent critical states, named combinatorial state, describing each a particular set of MDS:

- F-I combinatorial state defines an emergency in which a person who has fallen remains inert thereafter, regardless of his final position;
- F-D combinatorial state defines an emergency in which a person who has fallen remains lying down on the ground thereafter;
- I-D combinatorial state defines an emergency in which a person is inert and lying down on the ground;

The man down situations are represented as a function of critical state occurrences, summed up in the set (F∩I)∪(F∩D)∪(I∩D). However, the F-I-D combinatorial state is already implied in the combinatorial states sets and will not be referred to herein. The I-D state may be counter-intuitive, but it is at the core of the project purpose to develop a robust solution since it is focused on post-fall disability state and can detect some particular cases where fall state has not been detected or situations where the worker goes down but do not fall according to the definition above, for example the worker might feel unwell and squat slowly before going to the ground.

### 2.6. Detection Algorithms

The extreme value signals from the inertial measurements processing constitute the detection strategy variables in regards to the fall, immobility and down position states characterization. The detection strategy consists of several processing and analysis stages in order to train the algorithm and predict the critical states occurrence. The training phase begins with the building of statistical distribution models of extreme values of feature signals, segmented by their respective optimally-sized time windows. Then, the optimal threshold for the detection of the critical states is based on the analysis of the fusion of the detection probability provided by the feature signals statistical models. At last, the F-I, F-D and I-D combinatorial states are obtained by applying a simple logic AND function on pairs of detected critical states considering the signal segmentation as well by optimal time window sizes. The prediction phase is the application of the detection strategy on independent data, based on the previous critical states characterization. From Equations (7) and (8), given an extreme value signal $E_{s}\left(t,\tau \right)$ of the feature signal s(t) segmented according to a time window size $\tau_{s}$, as well as a detection threshold $\gamma_{s}$, the detection probability can be found by

(11) $$P_{D}=\mathrm{Pr}\left\{E_{s}\left(t,\tau \right)\begin{array}{c} \max\\ ⩾\\ ⩽\\ \min \end{array}\gamma_{s}|H_{1}\right\}$$

(12) $$P_{\mathrm{FA}}=\mathrm{Pr}\left\{E_{s}\left(t,\tau \right)\begin{array}{c} \max\\ <\\ >\\ \min \end{array}\gamma_{s}|H_{0}\right\}$$

where the detection condition differs depending on the related extremum, the minima (min) or maxima (max) of the extreme values signal. Each optimal threshold and time window size values are determined by maximizing the MCC through the performance analysis.

#### 2.6.1. Fall Detection

Considering the proposed fall state definition from Section 2.5, the fall detection is based on the analysis of extreme value signals, as such, the mimima and maxima of the mean of the acceleration norm $\bar{A}\left(t\right)$, the maxima of the mean of the rotational speed norm $\bar{W}\left(t\right)$ and the maxima of the mean of the derivative of the tilt angle $\bar{\dot{\rho }}\left(t\right)$. The extreme values of these feature signals are analyzed and studied through the database fall scenarios, applying time segmentation by specific time window sizes which differ according to the transient nature of signals. Therefore, the fall detection features signals are given by

(13) $$E_{F}\left(t,\tau_{F}\right)=\left[\begin{array}{c} E_{\bar{A}}^{\min}\left(t,\tau_{\bar{A}}^{\min}\right)\\ E_{\bar{A}}^{\max}\left(t,\tau_{\bar{A}}^{\max}\right)\\ E_{\bar{W}}^{\max}\left(t,\tau_{\bar{W}}^{\max}\right)\\ E_{\bar{\dot{\rho }}}^{\max}\left(t,\tau_{\bar{\dot{\rho }}}^{\max}\right) \end{array}\right]=\left[\begin{array}{c} \min\left(\bar{A}\left[t,t+\tau_{\bar{A}}^{\min}\right]\right)\\ \max\left(\bar{A}\left[t,t+\tau_{\bar{A}}^{\max}\right]\right)\\ \max\left(\bar{W}\left[t,t+\tau_{\bar{W}}^{\max}\right]\right)\\ \max\left(\bar{\dot{\rho }}\left[t,t+\tau_{\bar{\dot{\rho }}}^{\max}\right]\right) \end{array}\right]$$

where $\tau_{F}$=${\left[\tau_{\bar{A}}^{\min}\;\tau_{\bar{A}}^{\max}\;\tau_{\bar{W}}^{\max}\;\tau_{\bar{\dot{\rho }}}^{\max}\right]}^{T}$ are the time window sizes. Since the feature signals transients do not necessarily coincide in time, the fusion function is defined as the product of the maximum detection probabilities from the individual extreme values analysis over a common time segmentation, as

(14) $$L_{F}\left(E_{F}\left(t,\tau_{F}\right),\tau_{F,L}\right)=\prod_{i=1}^{M_{F}}\frac{\max({\mathrm{pdf}}_{i}\left(E_{F,i}\left[t,t+\tau_{F,L}\right]\right))}{{\mathrm{pdf}}_{i,\max}}$$

where $M_{F}$ is the number of feature signals and $\tau_{F,L}$ is the time window size. In order to standardize the weight of each detection probabilities in the fusion process, the probability density function are normalized to a unit scale by respectively dividing by the maximum probability value ${\mathrm{pdf}}_{i,\max}$. The expression of the fall detection signal $y_{F}$ is defined as

(15) $$y_{F}\left(t\right)=\left\{\begin{array}{ccc} 0 & \mathrm{if} & L_{F}\left(E_{F}\left(t,\tau_{F}\right),\tau_{F,L}\right)\leq \gamma_{F}\\ 1 & \mathrm{if} & L_{F}\left(E_{F}\left(t,\tau_{F}\right),\tau_{F,L}\right)>\gamma_{F} \end{array}\right.$$

where $\gamma_{F}$ is the fall detection threshold.

#### 2.6.2. Immobility Detection

Considering the proposed immobility critical state definition from Section 2.5, the immobility state detection is based on observation of minimal body movements by analyzing the extreme value signals, as such, the minima of the variance of the acceleration norm $\sigma_{A}^{2}\left(t\right)$, the minima of the variance of the rotational speed norm $\sigma_{W}^{2}\left(t\right)$ as well as the minima of the variance of the derivative of the tilt angle $\sigma_{\dot{\rho }}^{2}\left(t\right)$. The use of signals variance is more suitable for low level activity detection, ensuring that detection properties persist over time as these signals low amplitudes may drift over time and compromise the detection. Therefore, the immobility detection features signals are given by

(16) $$E_{I}\left(t,\tau_{I}\right)=\left[\begin{array}{c} E_{\sigma_{A}^{2}}^{\min}\left(t,\tau_{\sigma_{A}^{2}}^{\min}\right)\\ E_{\sigma_{W}^{2}}^{\min}\left(t,\tau_{\sigma_{W}^{2}}^{\min}\right)\\ E_{\sigma_{\dot{\rho }}^{2}}^{\min}\left(t,\tau_{\sigma_{\dot{\rho }}^{2}}^{\min}\right) \end{array}\right]=\left[\begin{array}{c} \min\left(\log_{10}\sigma_{A}^{2}\left[t,t+\tau_{\sigma_{A}^{2}}^{\min}\right]\right)\\ \min\left(\log_{10}\sigma_{W}^{2}\left[t,t+\tau_{\sigma_{W}^{2}}^{\min}\right]\right)\\ \min\left(\log_{10}\sigma_{\dot{\rho }}^{2}\left[t,t+\tau_{\sigma_{\dot{\rho }}^{2}}^{\min}\right]\right) \end{array}\right]$$

where $\tau_{I}$=${\left[\tau_{\sigma_{A}^{2}}^{\min}\;\tau_{\sigma_{W}^{2}}^{\min}\;\tau_{\sigma_{\dot{\rho }}^{2}}^{\min}\right]}^{T}$ are the time window sizes. Since the immobility state is constant and non-transitory, the fusion function is defined as the product of the average detection probabilities from the individual extreme values analysis over a common time segment, as

(17) $$L_{I}\left(E_{I}\left(t,\tau_{I}\right),\tau_{I,L}\right)=\prod_{i=1}^{M_{I}}\frac{\mathrm{mean}({\mathrm{pdf}}_{i}\left(E_{I,i}\left[t,t+\tau_{I,L}\right]\right))}{{\mathrm{pdf}}_{i,\max}}$$

where $M_{I}$ is the number of feature signals and $\tau_{I,L}$ is the time window size of the feature signals fusion. The same scaling operation from Equation (14) is apply to standardize each probability weights. The expression of the immobility detection status signal is defined as

(18) $$y_{I}\left(t\right)=\left\{\begin{array}{ccc} 0 & \mathrm{if} & L_{I}\left(E_{I}\left(t,\tau_{I}\right),\tau_{I,L}\right)\leq \gamma_{I}\\ 1 & \mathrm{if} & L_{I}\left(E_{I}\left(t,\tau_{I}\right),\tau_{I,L}\right)>\gamma_{I} \end{array}\right.$$

where $\gamma_{I}$ is the immobility state detection threshold.

#### 2.6.3. Down Detection

The body tilt angle variable is commonly used in fall detection algorithms to eliminate most of false positive results, by monitoring the vertical (0${}^{\circ }$ angle) to horizontal transition of the body orientation, where the post-impact stage of fall event is defined by a critical tilt angle value [18,19]. The measurement of the horizontal position angle is not bounded at 90${}^{\circ }$ since the position of the device, the topography of the terrain and the way the body lay down are some examples of factors where the angle value could be greater. Considering that a MDS does not necessarily involve a fall, down position state is, as proposed, an independent critical state. The down position state detection is based on the analysis of extreme value signal, such as, the maximum of mean of the tilt angle $\bar{\rho }\left(t\right)$ over a specific time segment. Therefore, the down detection feature signal are given by:

(19) $$E_{D}\left(t,\tau_{D}\right)=\left[\begin{array}{c} E_{\bar{\rho }}^{\max}\left(t,\tau_{\bar{\rho }}^{\max}\right) \end{array}\right]=\left[\begin{array}{c} \max\left(\bar{\rho }\left[t,t+\tau_{\bar{\rho }}^{\max}\right]\right) \end{array}\right]$$

where $\tau_{D}$ = $\left[\tau_{\bar{\rho }}^{\max}\right]$ is the time window size. The interpretation of $E_{\bar{\rho }}^{\max}$ data can be altered by several unknown factors such as ground level, infrastructures, etc. Thus, the down position detection threshold is chosen by setting the type II error rate to 1% or $P_{D}=0.99$. The function of down position state $y_{D}\left(t\right)$ is defined by

(20) $$y_{D}\left(t\right)=\left\{\begin{array}{ccc} 0 & \mathrm{if} & E_{\bar{\rho }}^{\max}\left(t,\tau_{\bar{\rho }}^{\max}\right)\leq \gamma_{D}\\ 1 & \mathrm{if} & E_{\bar{\rho }}^{\max}\left(t,\tau_{\bar{\rho }}^{\max}\right)>\gamma_{D} \end{array}\right.$$

where $\gamma_{D}$ is the down position state detection threshold.

#### 2.6.4. Man Down Detection

This study on man down situations solves the detection problem by generalizing these emergencies according to the combination of independent critical states occurrences, namely the combinatorial states. Indeed, based on the proposed global MDS definition in Section 2.5, the detection strategy comes down to detect at least two different critical states occurrences in a certain time frame to identify a MDS. The combinatorial states detection is defined by the logical fusion of pairs of independent critical state detection, basically a logical AND (∧) operation over ANY (⋁) critical states occurrence over specific time segmentation, as

(21) $$y_{F\text{-}D}\left(t\right)=⋁\left\{y_{F}\left[t,t+\tau_{F\text{-}D}\right]\right\}\wedge ⋁\left\{y_{D}\left[t,t+\tau_{F\text{-}D}\right]\right\}$$

(22) $$y_{F\text{-}I}\left(t\right)=⋁\left\{y_{F}\left[t,t+\tau_{F\text{-}I}\right]\right\}\wedge ⋁\left\{y_{I}\left[t,t+\tau_{F\text{-}I}\right]\right\}$$

(23) $$y_{I\text{-}D}\left(t\right)=⋁\left\{y_{I}\left[t,t+\tau_{I\text{-}D}\right]\right\}\wedge ⋁\left\{y_{D}\left[t,t+\tau_{I\text{-}D}\right]\right\}$$

where $\tau_{F\text{-}D}$, $\tau_{F\text{-}I}$ and $\tau_{I\text{-}D}$ are time windows of each combinatorial state. Thus, the MDS prediction is defined as the inclusive disjunction of the combinatorial states, expressed as a logical OR (∨) operation over the combinatorial states detection signals, as

(24) $$y_{\mathrm{MDS}}\left(t\right)=y_{F\text{-}D}\left(t\right)\vee y_{F\text{-}I}\left(t\right)\vee y_{I\text{-}D}\left(t\right).$$

This algorithm ignores if F, I and D situations appear at the same time or after each others, but rather assess if they appear in a time window specific to each combinational state, and thus avoid to have to determine the beginning and the end of each critical state in order to assess if the states do indeed follow each other. The actual sequence of events does not provide such significant data for our algorithm, where only the presence of a critical state is required for the combination assessment. Moreover, such approach simplifies the algorithm as it avoids the characterization of the timing or interleaving of F-I and F-D.

### 2.7. Workers Physical Test Protocol

In order to validate the MDS detection strategy and the solution implementation within the CRITIAS digital earpiece, a formalized physical test protocol is proposed. This also allows to test the developed detection algorithms using head movements, which differ from inertial measurements from IMU positioned at the waist as in *SisFall* database. Moreover, the scenarios created by state-of-the-art protocols used in fall detection studies are not suitable for workers typical activities. Thus, the proposed physical tests are designed to mimic some ADL and typical workers activities that highlight extreme cases and frequent false alarms situations as well as test scenarios involving the critical states F, I and D, executed in a controlled environment.

**Figure 1:**
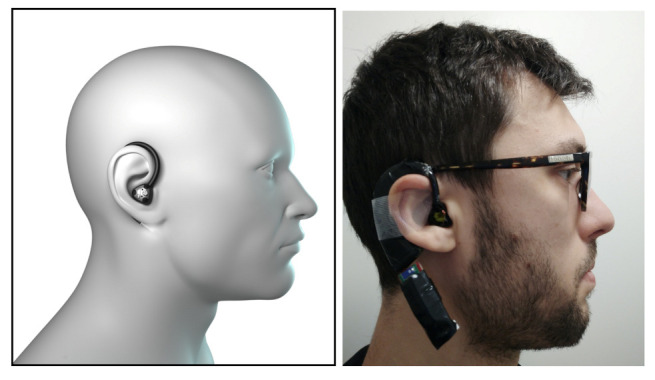
Digital earpiece prototype.

The earpiece prototype used a STMicroelectronics LSM6DS3 IMU. The setup of the digital earpiece prototype is shown in Figure 1. A *Bluetooth* wireless module enables the IMU data transmission to a computer for post-processing purpose. The inertial data from the IMU was sampled at 100 Hz, which is half frequency used by the reference *SisFall* database.

The proposed physical tests protocol, described in Table 1, includes tests that have already been used in protocols from other fall detection studies [20,21,22] and were also inspired from firefighter’s fitness assessment test [23]. The equipment used to perform the physical tests are: a chair, a flight of stairs, a mattress (≥0.75 m thick), a stick (1.5 m); a ball (0.30 m diameter, 10 kg); and a sled (20 kg).

## 3. Results

### 3.1. *SisFall*-Models and Validation

**Figure 2:**
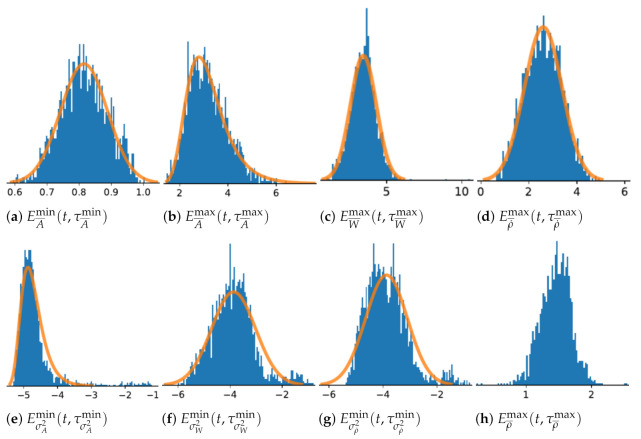
Distributions of extreme values signals.

The fall scenarios from the *SisFall* database is primary used to characterize the distributions of each extreme values signals use for each critical states detection algorithms presented in previous section. Figure 2 presents the distributions and the estimated statistical models obtained by the optimization of MCC, see Equation (10), through time windows size parametrization analysis, while the different models parameters and optimal time windows sizes are given in Table 2.

**Figure 3:**
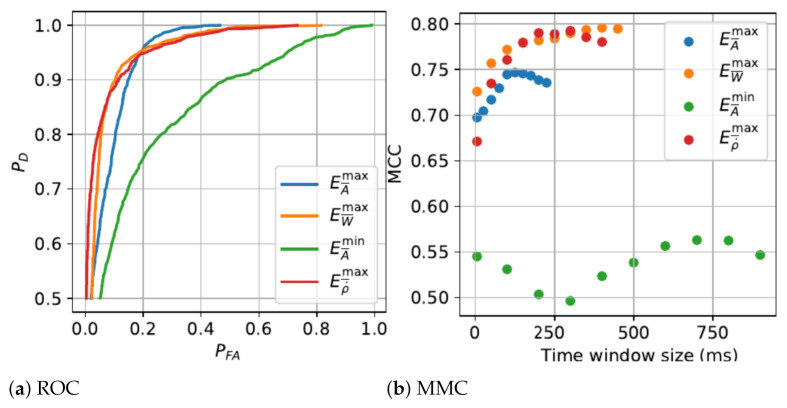
Fall features analysis.

**Figure 4:**
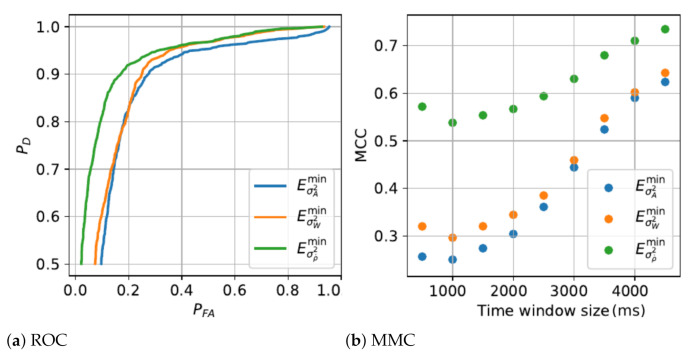
Immobility features analysis.

**Figure 5:**
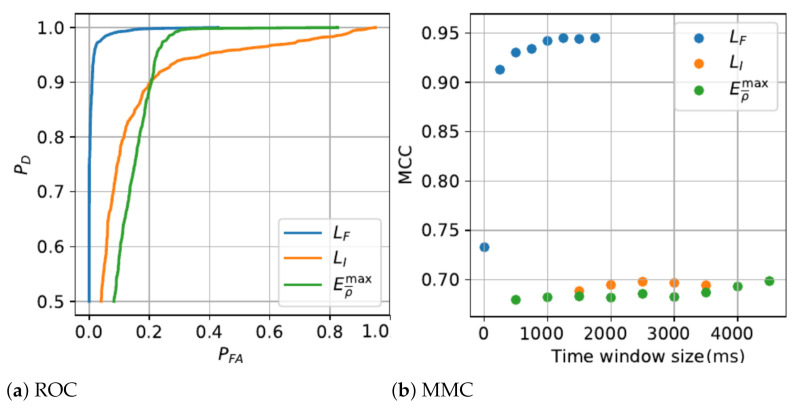
Features fusion analysis.

The detection algorithms performance and the parametric analysis from the training phase are presented in Figure 3, Figure 4 and Figure 5. Table 3 shows the performance results of critical states, combinatorial states and MDS detection on independent tests using a 10-fold cross validation method.

### 3.2. Workers Physical Tests-Validation

In order to validate that the algorithm developed using the *SisFall* database can be used for monitoring worker’s activities with the in-ear device solution, three volunteers (males between 21 and 25 years old) performed 129 physical tests (92 tests of ADL scenarios and 37 tests simulating MDS scenarios) based on the proposed workers application-based physical test protocol from Table 1. The summary of MDS and critical states detection results are shown in Table 4.

## 4. Discussion

### 4.1. *SisFall*-Models and Validation

Results of fall features signals analysis demonstrates that the transient of extreme values signal $E_{\bar{A}}^{\max}$ is by far the shortest with an optimal 125 ms (25 × 5 ms/sample) time window size. Other extreme value fall features signals $E_{\bar{\dot{\rho }}}^{\max}$, $E_{\bar{W}}^{\max}$ and $E_{\bar{A}}^{\min}$ have much longer optimal time window size, respectively of 300 ms, 405 ms and 730 ms. Fall detection based on $E_{\bar{A}}^{\max}$ feature score the best detection rate, up to 95%, but also generated the highest false positive rate. Considering the MCC, $E_{\bar{\dot{\rho }}}^{\max}$ had the best performance principally due to lowest false positive rate, making it the most relevant for fall state detection. The global fall scenarios prediction results over the *SisFall* database show persistent scores with an average accuracy up to 97%. This rate is congruent with other study [24], which used a same level detection method and tested with same database.

Since the immobility state is based on non-transitory features, the detection certainty and detection performance increase with time window size, thus the optimal time window for each immobility state features is 4.5 s, the longest window studied considering the finite length of database inertial measurement records. Immobility state features show similar detection performances although $E_{\sigma_{\dot{\rho }}^{2}}^{\min}$ is slightly ahead with higher precision rate and MCC. In the case of the minima of the variance of derivative of the tilt angle (Figure 2g), neither Normal or Gumbel distribution were offering a perfect fit. The statistical test based on the logarithm RML of functions of normal over Gumbel distributions, as discussed at Section 2.3, was marginally leaning toward the normal distribution. Besides, the maxima of the derivative of the tilt angle (Figure 2d) was best fitted by the normal distribution. These two factors directed us toward utilising the normal distribution.

For down position state detection, $E_{\bar{\rho }}^{\max}$ feature has also non-transient characteristics, more distinctive with a longer time window, but has a significant error detection rate explained by the large tilt angle positions of some ADL scenarios. The average value of $E_{\bar{\rho }}^{\max}$ distribution is 1.451 ± 0.003 radian (≈83${}^{\circ }$), which is a little less than expected value for a horizontal position. The tilt angle threshold is set at 0.87 radian, or approximately 50 degrees, which sets the detection rate at 99% for down position state based on fall scenarios of the database. The down position state is a good indicator of emergency occurrence despite a 28.2% false alarm rate, issue that is mitigated by critical states detection fusion. Indeed, the F-D combinatorial state has by far the best potential of MDS detection with an accuracy rate exceeding 98% and despite detection rates of combinatorial states are slightly lower than individual critical state detection rates, false positive rates are significantly reduced to well below 1%. Critical state fusion is then essential to the reduction of false alarms rates and related undesirable impacts (loss of time, loss of confidence and costs), which are the main causes of insufficient deployment of MDS detection systems in geriatric practice and industrial sectors [3]. The effectiveness and reliability of the MDS detection strategy, based on the proposed global and straightforward definition, is demonstrated by impressive overall prediction performance, with accuracy and detection rates over 99% and an 1.1% false alarm rate. These results are confirming our hypothesis and are very encouraging when compared to others, in practice surpassing most results from the similar inertial-based and sensor fusion fall detection solutions (accelerometer, gyroscope or both) [25,26].

### 4.2. Workers Physical Tests-Validation

The preliminary results from tests based on the proposed workers application-based physical test protocol show that time window size is a critical factor for the immobility state detection, mainly in order to reduce false positive. Also, even if inertial data was captured from a different location than data from *SisFall* database used for the training phase, i.e., the head instead of the waist, the fall detection performed well, correctly detecting 20 fall scenarios out of 24. This confirms the adaptability of the proposed detection strategy, given that only six false positive results of the fallen state were detected, all of which occurred during ADL scenarios involving jumps and high velocity motions. Otherwise, most of critical states detected over ADL scenarios tests were immobility and down position. The potential MDS false alarms were mostly rejected by the fusion algorithm and detection method since only a few critical state detection occurred during the same time window segmentation. Indeed, only two ADL scenario executions were wrongly classified as MDS, a meager 2.2% false alarm rate, both during a test where the subjects lie down on the ground for a few seconds, which led to misinterpreting these situations as I-D states. These results confirm that the detection fusion function reduces the MDS false alarms conditions compared to the individual critical states detection. Thus, combinational states and MDS detection results also indicate a good overall detection performance with a detection rate of 81.1% and a 93% accuracy. Over the years, many similar realizations using in-ear or head-level inertial sensors has been published ([27,28,29]) but theirs targets is mainly the elder population which reduce the relevance of comparing the results of the experimental tests carried out. Globally, these preliminary results are also encouraging considering the use of training data from waist movement which doubtlessly count for major part of the loss in performance with the in-ear sensor using the same algorithms.

## 5. Conclusions

Most research conducted on these types of detection methods have characterized the man down situations by a specific and isolated event such as a fall or the worker’s immobility, thus achieving a high detection rate of the specific event, but with somewhat more limited performances as to detecting actual man down situations.

This study has broadened the characterization of MDS into various combinations of distinct events, expressed as critical states: a fall (F), lying on the ground (D) and prolonged immobility (I); whereby any one of these critical states, if taken independently, does not fully characterize a MDS. Each critical state is the logical output of a simple detection strategy of its related event, based on an optimally calibrated threshold, which provides the best possible detection rate of the event. A relatively simple decision-making strategy, where these critical states are combined through logical fusion, can achieve a very high detection rate and a very low false alarm rate. The overall result is an integrated solution, using digital earpiece designed by the CRITIAS Chair, for the hearing protection of workers and an efficient man down situation detection device.

An enhanced physical tests protocol including additional MDS scenarios is needed to continue the detection algorithms training and validation process. Ideally, future work should focus on making an initial database using the earpiece on real workers from real work environment to analyze the actual worker’s movements and critical situations. The solution could also be integrated to digital earpieces for hearing aids and thus detect elderly falls, which are a larger-scale problem given that the elderly population tends to live alone and is more vulnerable to falls.

For optimization purposes, using larger sets of training data and more advanced machine learning methods such as neural networks and deep learning might be applied to the proposed detection strategy. The digital earpiece integrating the inertial platform could be enhanced by a left-ear right-ear strategy in order to add data redundancy and correlation between off-centered devices. Monitoring the worker’s vital signs has also been proposed in the literature for the early assessment of health problems, thus, the CRITIAS Chair is developing acoustic methods to measure vital signs with the digital earpiece. The instrumentalized digital earpieces could be used as a “black box” recording relevant information to understand causes of accidents or other events, similarly to the devices used on board aircraft. 

## Figures and Tables

**Table 1:** Workers physical test protocol.

#	Description	Chair	Stairs	Mattress	Stick	Ball	Sled
1	Take a Ground Object						
2	Long Bend (1 time)						
3	Lean repeatedly (5 times)						
4	Lie down on the back						
5	Lie on the floor on your stomach						
6	Lie on the ground on the right side						
7	Lie on the ground on the left side						
8	Sit on a chair						
9	Stay						
10	Fall forward						
11	Fall backward						
12	Walk (20 m)						
13	Run (20 m)						
14	Alternate walk-run (40 m)						
15	Cough						
16	Up, down stairs (10 steps)						
17	Jump on the spot						
18	Jump from the top of a chair						
19	Jump a length without momentum						
20	Jump a length with momentum						
21	Roll						
22	Ground Crawl						
23	Roll a ball while moving						
24	Roll a ball back						
25	Push a sled to weight						
26	Hammer with two hands						

**Table 2:** State features detection characterization.

Signal	Distribution	Locality	Scale	Window Size (ms)	Threshold
$E_{\bar{A}}^{\min}$	Normal	μ = 0.821 ± 0.010	σ = 0.0711 ± 0.0033	730 ± 50	0.8590 ± 0.0090 g
$E_{\bar{A}}^{\max}$	Gumbel	*u* = 2.81 ± 0.18	β = 0.699 ± 0.076	125 ± 15	2.08 ± 0.14 g
$E_{\bar{W}}^{\max}$	Normal	μ = 3.435 ± 0.045	σ = 0.850 ± 0.021	405 ± 15	2.432 ± 0.058 $\frac{\mathrm{rad}}{s}$
$E_{\bar{\dot{\rho }}}^{\max}$	Normal	μ = 2.6039 ± 0.0059	σ = 0.7816 ± 0.0051	300	1.682 ± 0.018 $\frac{\mathrm{rad}}{s}$
$E_{\sigma_{A}^{2}}^{\min}$	Gumbel	*u* = −4.8790 ± 0.0032	β = 0.2751 ± 0.0046	4500	$10^{-4.427\pm 0.039}$ $g^{2}$
$E_{\sigma_{W}^{2}}^{\min}$	Normal	μ = −3.8673 ± 0.0088	σ = 0.8483 ± 0.0084	4500	$10^{-2.920\pm 0.074}$ $\frac{{\mathrm{rad}}^{2}}{s^{2}}$
$E_{\sigma_{\dot{\rho }}^{2}}^{\min}$	Normal	μ = −3.8721 ± 0.0072	σ = 0.7719 ± 0.0060	4500	$10^{-3.1509\pm 0.0023}$ $\frac{{\mathrm{rad}}^{2}}{s^{2}}$

**Table 3:** States detection prediction results.

State	$P_{D}$	$P_{\mathrm{FA}}$	Window (ms)	MCC	Accuracy	Threshold
F	0.966 ± 0.017	0.0222 ± 0.0068	1475 ± 220	0.944 ± 0.019	0.9732 ± 0.0090	0.0254 ± 0.0030
I	0.828 ± 0.033	0.135 ± 0.023	2650 ± 335	0.690 ± 0.026	0.850 ± 0.013	0.038 ± 0.014
D	0.9889 ± 0.0059	0.2822 ± 0.0074	4500	0.6979 ± 0.0091	0.8260 ± 0.0051	0.0038 rad
F-D	0.955 ± 0.016	0.0011 ± 0.0018	4800 ± 1160	0.962 ± 0.012	0.9814 ± 0.0060	N/A
F-I	0.794 ± 0.031	0.0037 ± 0.0042	7500	0.830 ± 0.026	0.915 ± 0.013	N/A
I-D	0.815 ± 0.034	0.0066 ± 0.0059	3850 ± 240	0.843 ± 0.029	0.922 ± 0.015	N/A
MDS	0.9944±0.0037	0.0107 ± 0.0053	N/A	0.9825 ± 0.0080	0.9909 ± 0.0037	N/A

**Table 4:** Summary of workers physical tests results.

State	Nb. of MDS	Nb. of ADL	TP	FP	$P_{D}$	$P_{\mathrm{FA}}$	MCC	Accuracy
F-D	24	92	17	0	0.708	0.0	0.811	0.940
F-I	24	92	18	0	0.75	0.0	0.839	0.948
I-D	37	92	25	2	0.676	0.022	0.727	0.891
MDS	37	92	30	0	0.811	0.022	0.826	0.930

## Abbreviations

The following abbreviations are used in this manuscript:

MDS	Man Down Situations
F	Fall state
I	Immobility state
D	Down position state
ADL	Activities of Daily Living
IRSST	Institut de Recherche Robert-Sauvé en Santé et en Sécurité du travail
CRITIAS	EERS-CRSNG Industrial Research Chair in In-Ear Technologies
IMU	Inertial Measurement Unit
MEMS	Micro-Electromechanical System
TP	True Positive
FP	False Positive
TN	True Negative
FN	False Negative
MCC	Matthews Correlation Coefficient
ROC	Receiver Operating Characteristic
RML	Ratio of Maximized Likelihood
